# Early administration of adalimumab for paediatric uveitis due to Behçet’s disease

**DOI:** 10.1186/s12969-019-0333-6

**Published:** 2019-06-10

**Authors:** Tomona Hiyama, Yosuke Harada, Takehiko Doi, Yoshiaki Kiuchi

**Affiliations:** 1 Department of Ophthalmology and Visual Science, Graduate School of Biomedical Sciences, Hiroshima University, 1-2-3 Kasumi, Minami-ku, Hiroshima, 734-8551 Japan; 2 Department of Paediatrics, Hiroshima University, 1-2-3 Kasumi, Minami-ku, Hiroshima, 734-8551 Japan

**Keywords:** Adalimumab, Behçet’s disease, Paediatrics, Uveitis, TNFα antagonist

## Abstract

**Background:**

Behçet’s disease is a chronic inflammatory multisystem disorder that is characterised by oral and/or genital ulcerations as well as intraocular inflammation. Recurrent retinal vasoocclusive episodes and macular involvement may lead to severe loss of visual acuity. Patients may eventually become resistant to systemic corticosteroid and develop side effects; therefore, other immunosuppressive therapies are needed. Biologic agents are promising for the treatment of Behçet’s disease-associated uveitis. Here, we report two cases of paediatric uveitis due to Behçet’s disease that were successfully treated by early administration of adalimumab.

**Case presentation:**

Patient 1 was an 11-year-old girl who presented with right conjunctival injection and photophobia. Patient 2 was a 14-year-old girl who presented with blurry vision in the left eye. Both patients were treated with topical treatment and prednisolone for uveitis; however, relapses occurred during the tapering of prednisolone. The patients were diagnosed with Behçet’s disease, and adalimumab therapy was initiated. In both cases, the inflammation was well-controlled by adalimumab administration without local or systemic corticosteroid.

**Conclusions:**

Adalimumab is effective for treating children with Behçet’s disease-associated uveitis. Control of ocular inflammation was achieved without local and systemic corticosteroid, thus preventing further complications.

## Background

Behçet’s disease is a chronic inflammatory multisystem disorder that is characterised by oral and/or genital ulcerations as well as recurrent intraocular inflammatory episodes, which can be vision-threatening [1]. It may also involve the skin, joints, gastrointestinal tract, blood vessels, central nervous system, and other parts of the body [1]. The onset of Behçet’s disease typically occurs among individuals aged 20–40 years. Behçet’s disease is rarely observed in children or patients above the age of 50; the disease starts in childhood in 4–26% of cases [2, 3]. Furthermore, the time to diagnosis may be lengthy in children due to a lack of symptoms at the time of presentation [4–6]. Ocular manifestation typically comprises a recurrent bilateral non-granulomatous uveitis, which occurs in 30–70% of Behçet’s patients [1, 7]. In the PEDBD cohort study, ocular symptoms were observed less frequently in children than in adults, with higher prevalence in males [8]. Anterior uveitis of Behçet’s disease is typically managed by topical treatment, such as corticosteroid and mydriatic agents. Inflammation in the posterior segment can cause irreversible damage and may lead to severe loss of visual acuity; thus, systemic treatment is required [9]. In the acute phase, Behçet’s disease responds well to systemic corticosteroid; however, patients may eventually become resistant to systemic corticosteroid treatment. Moreover, long-term administration of systemic corticosteroids may cause complications, such as cataract, glaucoma, and growth defects in children. Therefore, steroid-sparing immunosuppressive therapy is required. [10]. With conventional treatment, the risk of blindness from posterior eye involvement and uveitis complications is approximately 25% at 10 years after the onset [1, 11, 12]. Biologic agents are now promising for treatment of Behçet’s disease. In Japan, infliximab was the only approved drug for Behçet’s disease; adalimumab is a newly available steroid-sparing drug. Currently, neither infliximab nor adalimumab is approved for paediatric use.

Here, we report successful treatment of two cases of paediatric uveitis due to Behçet’s disease; both were treated with early administration of adalimumab.

## Case presentation

### Case 1

**Fig. 1:**
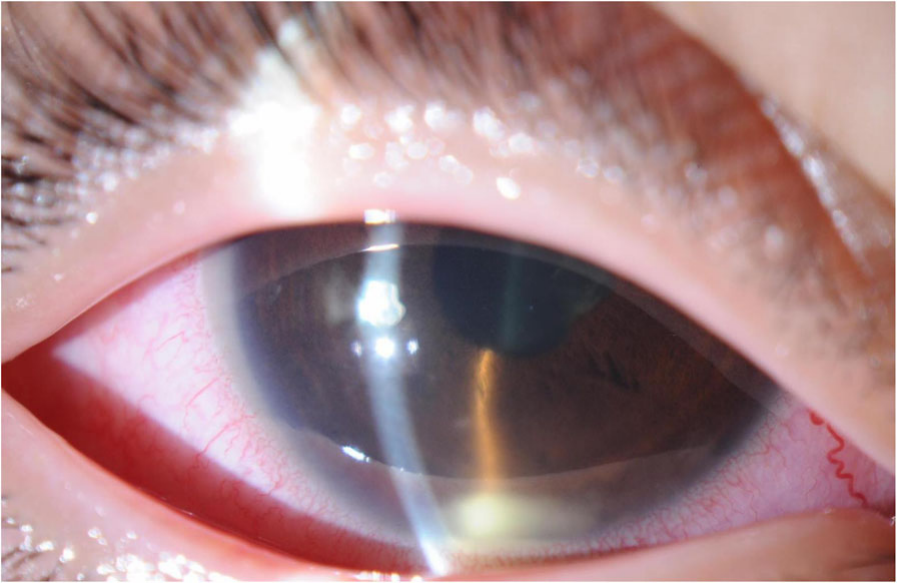
Clinical appearance of the right eye of Case 1 at presentation. Slit-lamp examination showed ciliary injection and diffuse non-granulomatous keratic precipitates in the right eye. Micro-hypopyon and anterior chamber cell grading of 3+ (based on the Standardization of Uveitis Nomenclature Working Group classification) were observed, as were posterior synechiae in the right eye

**Fig. 2:**
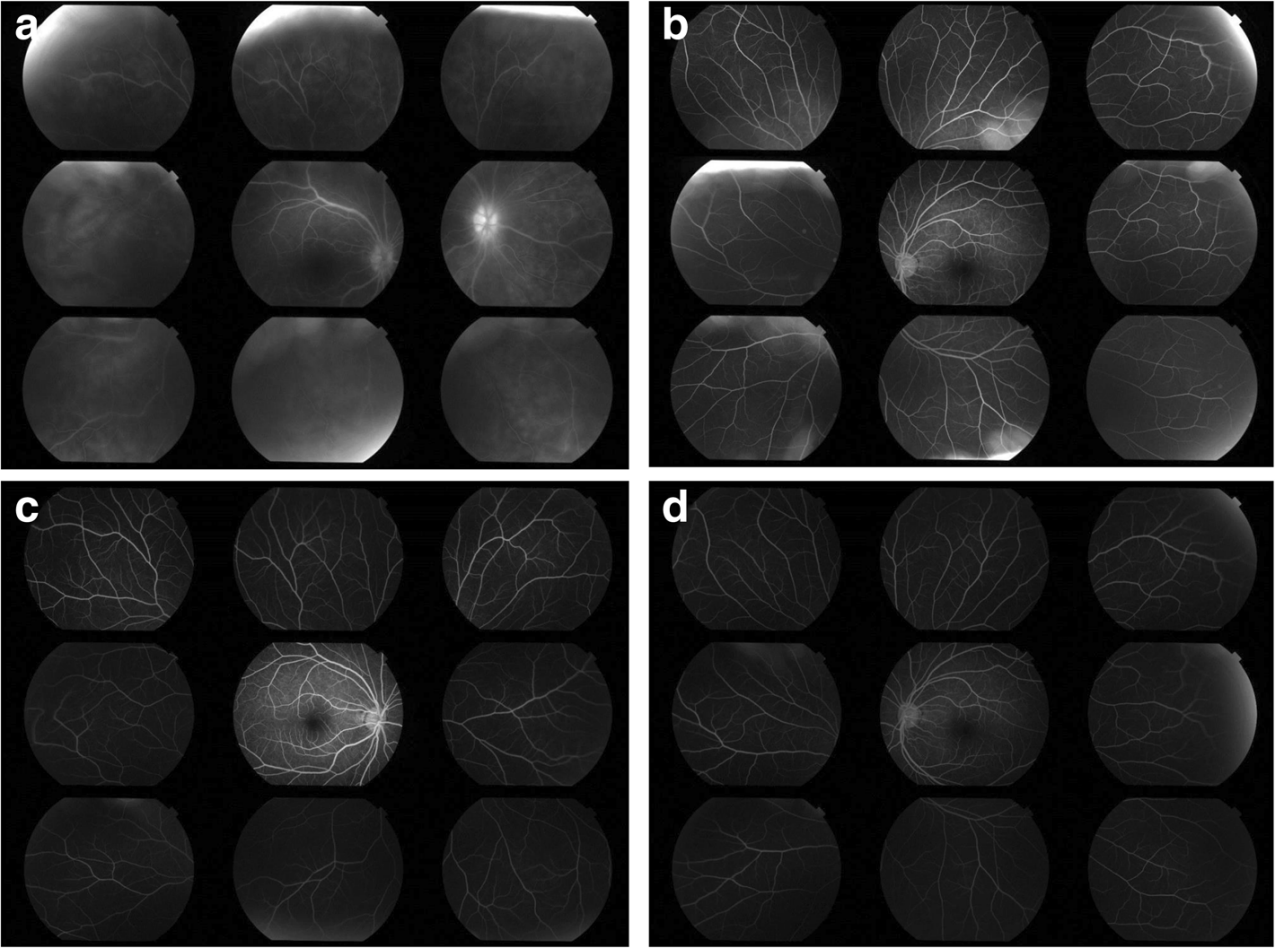
Fluorescein angiography (FA) of Case 1 before and after adalimumab treatment. **a**, **b** FA of the right and left eyes at presentation, respectively, revealed fern-like diffuse vascular leakage and optic disc hyperfluorescence in the right eye. FA of the left eye at presentation did not show any vasculitis. **c**, **d** FA of the right and left eyes at 5 months after adalimumab treatment, respectively. There were no signs of vasculitis in either eye

**Fig. 3:**
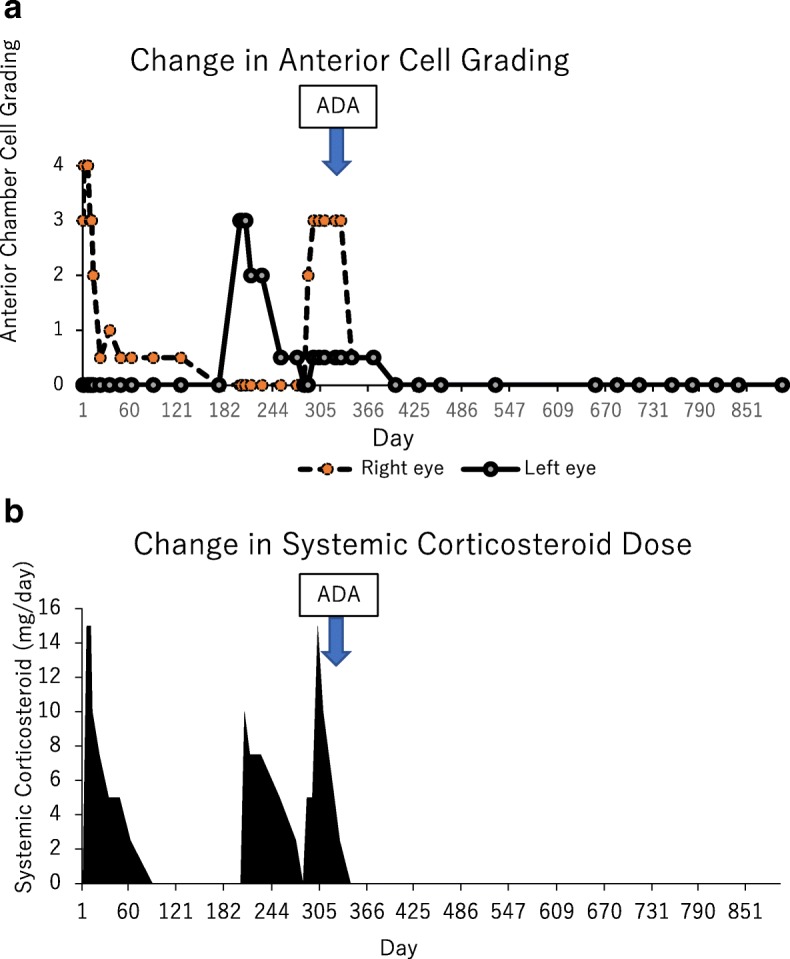
Changes in anterior chamber cell grade (**a**) and prednisolone dose (**b**) in Case 1. Anterior chamber cell grading in the right eye was 3+ (based on the Standardization of Uveitis Nomenclature Working Group classification) at presentation. After prednisolone 15 mg/day was initiated, ocular inflammation and best-corrected visual acuity (BCVA) gradually improved; however, relapse occurred in both eyes during tapering of prednisolone. Adalimumab was administered to avoid the side effects of systemic corticosteroid. BCVA improved to 1.5 in both eyes and the anterior chamber cell grade improved to less than 0.5+ within 2 weeks. Both local and systemic corticosteroid were discontinued, and complete control of inflammation was achieved with adalimumab

**Fig. 4:**
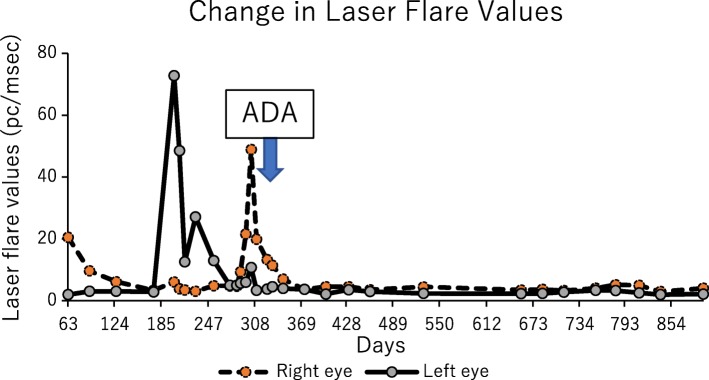
Change in laser flare photometry values in Case 1. Laser flare values were recorded from day 63. Increased laser flares were observed during two relapses under the influence of corticosteroid. After adalimumab was introduced, laser flare values dramatically improved and reached the normal range within 4 weeks, without local or systemic corticosteroid

An 11-year-old female patient with no previous history presented with right conjunctival injection and photophobia. The patient had previously been treated with fluorometholone 0.1% eye drops; however, the same symptoms recurred twice in 1 year. At presentation, her best-corrected decimal visual acuity (BCVA) was 0.4 in the right eye and 1.2 in the left eye. Intraocular pressures (IOPs) of the right and left eyes were 17 and 16 mmHg, respectively (Normal range: 10–21 mmHg). Slit-lamp examination showed ciliary injection and diffuse fine keratic precipitates. Micro-hypopyon and an anterior chamber cell grade of 3+ (based on the Standardization of Uveitis Nomenclature Working Group classification [13]) were observed; posterior synechiae were also present in the right eye. (Fig. 1) Fundus examination of the right eye was hazy and lacked clarity. The left eye exhibited no apparent abnormalities in the anterior chamber or fundus. Fluorescein angiography (FA) of the right eye revealed diffuse vascular leakage and optic disc leakage. (Fig. 2) The patient did not complain of arthralgia or genital ulcers, but had a history of recurrent oral ulcers. On the basis of these findings, the patient was diagnosed with unilateral panuveitis. The differential diagnosis was as follows: Behçet’s disease, juvenile idiopathic arthritis-related uveitis, HLA-B27-related uveitis, A20 haploinsufficiency, and sarcoidosis. Dexamethasone eye drops (0.1%, instilled hourly), tropicamide/phenylephrine eye drops (four times/day), 1% atropine eye drops (once/day), and prednisolone (15 mg/day orally) therapies were initiated for inflammation of the right eye. Further investigation revealed ileocecal ulcers and HLA-B51 positivity. Interferon-gamma release assay and tuberculin tests for tuberculosis infection, raid plasma regain assay, and *Treponema pallidum* antibody hemagglutination test for syphilis were negative; angiotensin-converting enzyme, antinuclear antibody, matrix metalloproteinase-3, and anti-citrullinated protein antibody levels were within the normal range. A20 haploinsufficiency was thought to be less likely in this patient, due to the absence of a family history of autoimmune diseases, genital ulcers, and fever spikes [14, 15]. In Behçet’s disease, oral ulcers heal without scars, uveitis typically involves the posterior chamber, and retinal vasculitis manifests with a fern-like pattern; in contrast, A20 haploinsufficiency is characterised by anterior uveitis [14]. Because of the presence of typical ocular symptoms and recurrent oral ulcers, the patient was diagnosed with the incomplete type of Behçet’s disease, in accordance with the Japanese diagnostic criteria for Behçet’s disease (revised in 1987) [16]. Inflammation in the anterior chamber and BCVA gradually improved after the treatment. However, a relapse occurred in the right eye and new-onset uveitis appeared in the left eye during the tapering of prednisolone. Adalimumab was administered subcutaneously to avoid the side effects of systemic corticosteroid. [17] The patient was 12-year-old and weighed 36 kg when adalimumab was started. Since there is no indication regarding the dose of adalimumab for paediatric Behçet’s disease, we administered 40 mg every 2 weeks without a loading dose, in accordance with the recommended dose for treatment of juvenile idiopathic arthritis-associated uveitis. The duration of uveoretinitis prior to starting adalimumab was 11 months. After beginning administration of adalimumab, the patient complained of transient abdominal pain, which resolved spontaneously. BCVA improved to 1.5 in both eyes and the anterior chamber cell grade improved to < 0.5+ within 2 weeks. (Fig. 3) Within 4 weeks, laser flare photometry values dramatically improved from the peak value of 48 ph/ms (physiological value approximately 3 ph/ms) in both eyes, to 3–4 ph/ms in the right eye and 2–3 ph/ms in the left eye. (Fig. 4) FA revealed improvement of retinal vasculitis. (Fig. 2) Oral ulcers healed without scars after adalimumab administration. Ileocecal ulcers were completely resolved on the follow-up colonoscopy, which was performed 3 months after initiation of the therapy. The inflammation has remained well-controlled by administration of adalimumab without local or systemic corticosteroid for 17 months. No side effects of adalimumab have been observed.

### Case 2

**Fig. 5:**
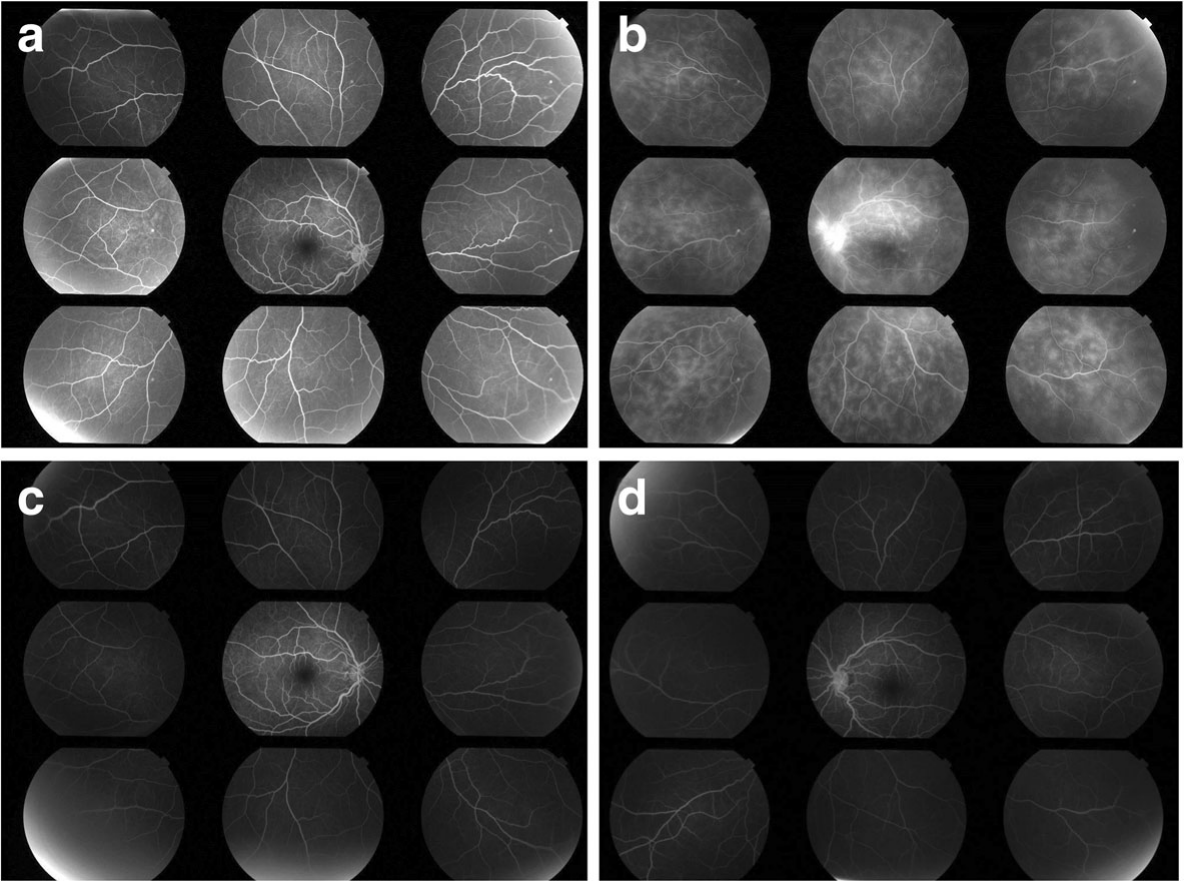
Fluorescein angiography (FA) of Case 2 before and after adalimumab treatment. **a**, **b** FA of the right and left eye at presentation, respectively, revealed fern-like diffuse vascular leakage and optic disc hyperfluorescence in the left eye. FA of the right eye at presentation did not show any vasculitis. **c**, **d** FA of the right and left eyes at 2 months after adalimumab treatment, respectively. There were no signs of vasculitis in either eye

**Fig. 6:**
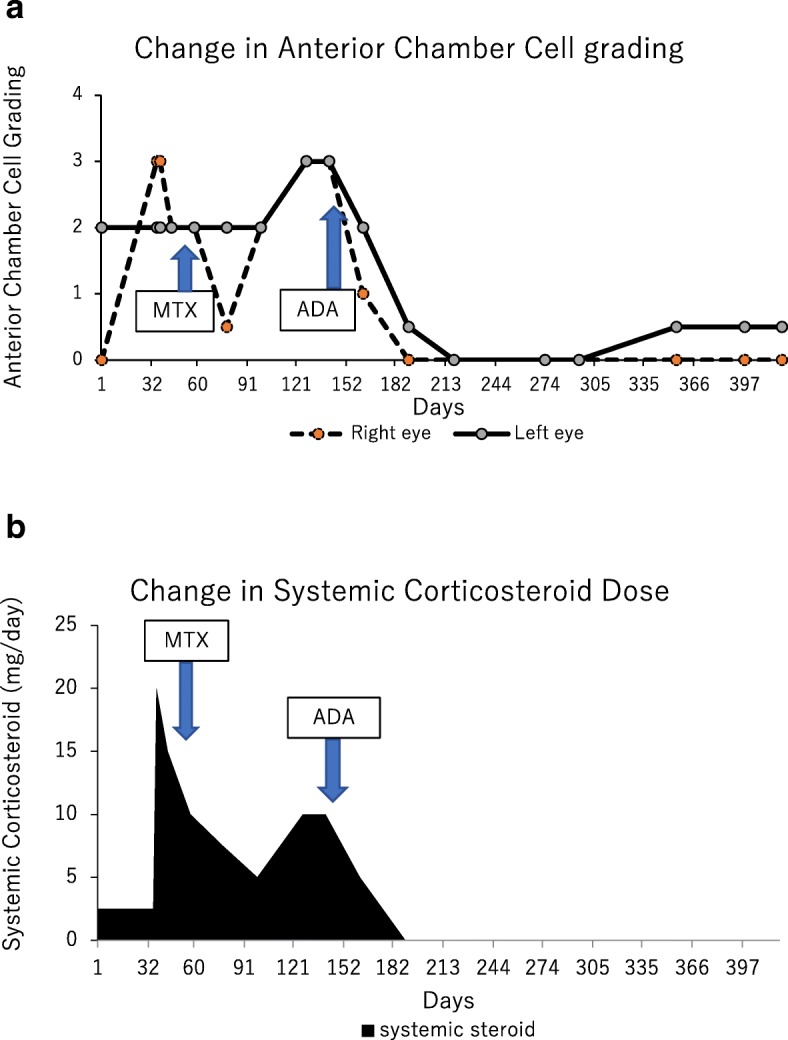
Changes in anterior chamber cell grade (**a**) and prednisolone dose (**b**) in Case 2. Retinal vasculitis recurred in both eyes, and prednisolone was increased to 20 mg/day, while methotrexate was initiated at 6 mg. A maximum of 12 mg methotrexate was administered; however, the patient experienced nausea and the inflammation relapsed. After the patient was diagnosed with an incomplete type of Behçet’s disease by the paediatrician, adalimumab was introduced, and prednisolone was slowly tapered. The anterior chamber cell grade dramatically improved from 3+ in both eyes to 0 and 0.5+ in the right and left eyes, respectively, within 7 weeks after beginning administration of adalimumab. The inflammation subsided, and local and systemic corticosteroid therapies were discontinued

**Fig. 7:**
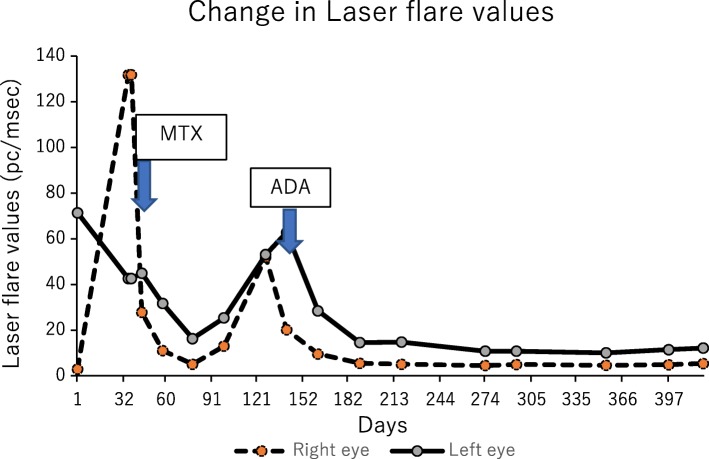
Change in laser flare photometry values in Case 2. Laser flare photometry values were recorded from day 1. Increased laser flare was observed during relapses. After adalimumab was introduced, laser flare values improved

A 14-year-old female patient reported blurry vision in the left eye for the past 8 months and had been diagnosed with uveitis at another clinic. Despite the administration of local and systemic corticosteroid, inflammation persisted; therefore, the patient was referred to our clinic. The patient presented with fine keratic precipitates and anterior chamber cell grade of 2+ in the left eye. The vitreous cell grade was 1+ in the right eye and 2+ in the left eye. FA showed diffuse fern-like capillary leakage and optic disc hyperfluorescence of the left eye. (Fig. 5) The BCVA was 1.2 in both eyes, and the IOPs of the right and left eyes were 16 and 22 mmHg, respectively. Non-ocular manifestations were oral ulcers and shoulder arthralgia. Skin or genital lesions were not observed. The differential diagnosis was as follows: Behçet’s disease, A20 haploinsufficiency, and idiopathic retinal vasculitis. Interferon-gamma release assay and tuberculin tests for tuberculosis infection, raid plasma regain assay, and *Treponema pallidum* antibody hemagglutination test for syphilis were negative; angiotensin-converting enzyme, antinuclear antibody, matrix metalloproteinase-3, and anti-citrullinated protein antibody levels were within the normal range. There was no family history of autoimmune diseases and colonoscopy revealed no abnormality. Behçet’s disease was suspected and the patient was referred to a paediatrician for further investigation. She tested negative for HLA-B51. Additionally, the following treatment (initiated in the previous clinic) was continued: 0.1% dexamethasone eye drops (four times/day), tropicamide/phenylephrine eye drops (once/day), and prednisolone (5 mg/day orally). In accordance with the Japanese diagnostic criteria for Behçet’s disease (revised in 1987), the patient was diagnosed with the incomplete type of Behçet’s disease on the basis of the presence of a typical ocular symptom and recurrent oral ulcers [16]. Retinal vasculitis recurred in both eyes; therefore, initiation of adalimumab was proposed to the patient; however, it was initially declined due to financial restrictions. Thus, prednisolone was increased to 20 mg/day, and methotrexate was initiated at 6 mg/day. (Fig. 6) A maximum of 12 mg methotrexate was administered; however, the patient experienced nausea and the inflammation relapsed. Subcutaneous adalimumab injection was then introduced, and prednisolone was slowly tapered. The patient was 15-year-old and weighed 53 kg when adalimumab was initiated, thus we administered 80 mg as a loading dose, followed by 40 mg every 2 weeks, starting 1 week after the loading dose, in accordance with the recommended dose for treatment of adult uveitis. The duration of uveoretinitis prior to starting adalimumab was 13 months. The anterior chamber cell grade improved from 3+ in both eyes to 0 and 0.5+ in the right and left eyes, respectively, within 7 weeks after beginning adalimumab administration. (Fig. 6) The peak values of laser flare photometry were 131.8 ph/ms in the right eye and 71.4 ph/ms in the left eye; these improved to 4–5 ph/ms and 10–12 ph/ms, respectively. (Fig. 7)  BCVA remained ≥ 1.5 in both eyes. Oral ulcers and arthralgia were improved after adalimumab therapy. The inflammation subsided, and local and systemic corticosteroid therapies were discontinued. The follow-up period of adalimumab was 12 months at the time this report was written, and the patient had not experienced any side effects.

## Discussion and conclusions

Here, we have reported successful treatment of paediatric uveitis due to Behçet’s disease via early administration of adalimumab. Extraocular symptoms, including oral ulcers, ileocecal ulcer, and arthralgia, were also improved by the therapy without systemic complications.

In Japan, anti-tumour necrosis factor- α (anti-TNF- α), infliximab was the only approved biologic agent for Behçet’s disease, despite the high prevalence of the disease. After adalimumab (anti-TNF- α) became available in 2016, the treatment strategy of non-infectious uveitis dramatically changed. Currently, neither infliximab nor adalimumab is approved for paediatric use; however, retrospective case series have shown the efficacies of both drugs in treatment of refractory non-infectious uveitis in children [18–20]. Importantly, the number of patients with Behçet’s disease is limited in many of the studies based on children. Deitch et al. reported the efficacy of adalimumab in 24 children with non-infectious uveitis, only four of which exhibited Behçet’s disease [21]. Ljubetic et al. and Biester et al. reported the efficacy of adalimumab for refractory childhood uveitis; however, Behçet’s patients were not included [22, 23]. The current case report shows the detailed clinical course of paediatric Behçet’s uveitis treated by adalimumab, which comprises valuable information for paediatricians and uveitis specialists.

Previous reports have shown the earlier initiation of anti-TNF- α for Behçet’s disease-associated uveitis results in better visual outcome and reduced frequency of ocular attacks [24, 25]. Guzelant et al. compared two groups and reported that those who were on other immunosuppressive treatment for a median of 26.5 months (9–50.5 months) prior to infliximab had better outcomes than those who were on other immunosuppressive treatment for a median of 60 months (25–84 months) [24]. Keino et al. also reported that the frequency of ocular attacks, severity of retinal vasculitis, and BCVA were significantly improved in a group of patients with a median duration of Behçet’s uveoretinitis of 15 months (11–18 months) prior to starting infliximab, compared with patients with a median duration of 89 months (40–112 months) [25]. Vallet et al. showed equal effectiveness of infliximab and adalimumab; thus, we suspected that early administration of adalimumab could also be beneficial [26]. In the present cases, the duration of uveoretinitis prior to starting adalimumab was approximately 11 months in case 1 and 13 months in case 2, which can be classified as “early.” Both patients achieved quiescence as a result of the early administration of adalimumab.

When treating uveitis in children, clinicians must consider the importance of preventing the complications of uveitis, as well as the side effects of treatment. The use of local corticosteroid is related to increased IOP, and the increased IOP is especially pronounced in children. Among patients with uveitis who were treated with topical difluprednate, there was reportedly an increase in IOP of more than 15 mmHg in 80% of children [27]. In both of our cases, we successfully tapered corticosteroid eye drops and systemic prednisolone after the administration of adalimumab. BCVA remained ≥ 1.5 and IOPs in both eyes were within the normal range. The patients have not experienced growth disorder or any other symptoms caused by administration of systemic corticosteroid.

We regularly measured the laser flare photometry values of both patients. Aqueous flare and cells are two parameters used as indicators of anterior chamber inflammation [28]. While the slit-lamp examination for assessment of intraocular inflammation remains subjective, laser flare photometry provides a noninvasive, objective, and quantitative measurement of aqueous humour protein levels in the anterior chamber [29]. Inflammatory mediators, such as tumour necrosis factor-alpha (TNF- α), are factors underlying the breakdown of the blood-aqueous barrier, which leads to elevation of laser flare [30]. The physiological laser flare value is approximately 3.0 ± 1.1 ph/ms in healthy individuals between 10 and 19 years of age [28]. Previous reports have shown that blood-aqueous barrier disruption is pronounced in Behçet’s disease; moreover, patients with high flare (≥ 20 ph/ms) tend to develop new complications, such as cataract, glaucoma, and posterior synechiae [31–33]. In the present cases, laser flare values decreased to normal to low flare range (< 20 ph/ms) and laser flare-derived complications did not develop with adalimumab treatment. [31, 33] Furthermore, in both cases, laser flare began to decrease after improvement of the anterior chamber cell grade. According to Holland et al., patients with low flare have a lower risk of vision loss or vision-threatening complications, regardless of the presence of high anterior chamber cell levels during the course of the disease [32]. The present cases emphasise the importance of laser flare monitoring, as treatment based on anterior cell grade alone may lead to loss of vision. FA taken after administration of adalimumab showed no sign of vascular leakage; furthermore, retinal structures remained intact in both cases. We managed to achieve complete control of inflammation due to adalimumab.

Adalimumab is not approved for treatment of paediatric ocular Behçet’s disease. However, we greatly desired to introduce adalimumab as treatment for these two patients, because we previously encountered a Behçet’s disease patient who had been treated with adalimumab after multiple severe recurrences. The patient showed no severe relapse after administration of adalimumab; however, BCVA improvement was limited due to irreversible retinal disruption, which largely involved the macula. The laser flare value of the patient remained high after quiescence, which may be due to irreversible disruption of the blood-ocular barrier. We learned from this patient that adalimumab should be introduced to uveitis patients before repeated inflammation causes irreversible structural and functional changes.

During adalimumab treatment, the patients in the present report did not experience any adverse effects. We chose to use adalimumab instead of infliximab, because we place great importance on stress-free, adequate, and continuous treatment, which results in minimal interference in the lifestyle of the affected children. Adalimumab can be self-administered and does not require hospitalisation or activity restriction; infliximab requires both of these accommodations. Moreover, adalimumab has a lower risk of anti-drug antibody formation [34].

Adalimumab may be effective in treating children with uveitis due to Behçet’s disease. Control of ocular inflammation and reduction of laser flare were achieved without local and systemic corticosteroid, thus preventing further complications. The visual outcome of Behçet’s disease has significantly improved since the introduction of biologic agents [35, 36]. We believe that it is essential to administer adalimumab before irreversible structural and functional changes occur. There are increased risks of serious infections, lymphoma, or other cancers as side effects of TNF- α antagonist administration. Long-term follow-up with a larger group of paediatric patients is necessary to clarify the efficacy and possible adverse effects of adalimumab.

## Data Availability

The datasets used and analysed during the current study are available from the corresponding author on reasonable request.

## Abbreviations

anti-TNF- α: Anti-tumour necrosis factor α
BCVA: Best corrected visual acuity
FA: Fluorescein angiography
IOP: Intraocular pressure
